# Tracking Inflammation in CAR-T Therapy: The Emerging Role of Serum Amyloid A (SAA)

**DOI:** 10.3390/cancers17193184

**Published:** 2025-09-30

**Authors:** Ilaria Pansini, Eugenio Galli, Alessandro Corrente, Marcello Viscovo, Silvia Baroni, Nicola Piccirillo, Patrizia Chiusolo, Federica Sorà, Simona Sica

**Affiliations:** 1 Dipartimento di Scienze di Laboratorio ed Ematologiche, Fondazione Policlinico Universitario A. Gemelli IRCCS, 00168 Rome, Italy; 2 Sezione di Ematologia, Dipartimento di Scienze Radiologiche ed Ematologiche, Università Cattolica del Sacro Cuore, 00168 Rome, Italy; 3 Dipartimento di Scienze Biotecnologiche di Base, Cliniche Intensivologiche e Perioperatorie, Università Cattolica del Sacro Cuore, 00168 Rome, Italy

**Keywords:** serum amyloid A (SAA), inflammatory biomarkers, acute-phase proteins (APPs), CAR-T cell therapy

## Abstract

**Background**: Chimeric antigen receptor (CAR) T-cell therapy has revolutionized treatment of relapsed/refractory large B-cell lymphoma (LBCL), but its administration is often complicated by cytokine release syndrome (CRS). Interleukin-6 (IL-6) is widely used to monitor CRS, though its clinical value diminishes after tocilizumab administration. We aimed to evaluate serum amyloid A (SAA), a dynamic acute-phase reactant, as a treatment-independent biomarker of inflammation and toxicity in CAR-T recipients. **Methods**: This retrospective study included 43 adults with LBCL treated with axicabtagene ciloleucel. SAA and other inflammatory markers were assessed from lymphodepletion through day +11 post-infusion. CRS and ICANS were graded per ASTCT criteria. Statistical analyses included Mann–Whitney U tests, Spearman’s correlation, and ROC curve analysis to evaluate predictive performance. **Results**: SAA levels peaked at day +4 and normalized by day +11, displaying wave-like kinetics. Levels were significantly higher in patients with any-grade CRS at early timepoints but showed no association with ICANS. SAA correlated strongly with CRP, suPAR, sST2, fibrinogen, ferritin, procalcitonin, and IL-6. Compared to IL-6, SAA was more predictive of CRS at day +2 and +4, and unaffected by tocilizumab. Baseline SAA also correlated with the mEASIX score, suggesting linkage to endothelial stress. Non-responders at 3-month PET had higher baseline SAA than responders (196.0 vs. 17.7 mg/L, *p* = 0.036), with ROC analysis yielding an AUC of 0.74 and an optimal threshold of 79.8 mg/L. **Conclusions**: SAA is a robust and dynamic marker of systemic inflammation, with potential utility in both toxicity monitoring and response prediction in the CAR-T setting. Its independence from IL-6 modulation positions it as a promising biomarker for future integration into clinical algorithms.

## Simple Summary

Serum amyloid A (SAA) is an inflammatory protein rapidly increasing during the acute phase in many conditions. In this study, we measured SAA levels in patients receiving CAR-T cell therapy for hematological malignancies, and examined how their correlation with cellular therapy-related inflammation and clinical outcomes. SAA levels increased shortly after infusion and were higher in patients who experienced cytokine release syndrome (CRS), a prevalent inflammatory side effect. Unlike interleukin-6 (IL-6), SAA remained a reliable and unbiased indicator of inflammation also after the administration of tocilizumab, a specific anti-CRS drug targeting IL-6-receptor. Interestingly, patients with high SAA levels prior to CAR-T were more likely to have poor responses at one and three months. These results suggest that SAA could be a useful tool for detection of toxicities and early prediction of resistance, helping to guide clinical decisions before or during CAR-T therapy.

## 1. Introduction

Chimeric antigen receptor (CAR) T-cell therapy has dramatically reshaped the treatment landscape for relapsed/refractory large B-cell lymphoma (LBCL), offering high response rates in otherwise difficult-to-treat populations. Those benefits may sometimes be obtained at the price of immune-mediated side effects, sometimes severe. The most frequent of these is cytokine release syndrome (CRS) [1,2].

Interleukin-6 (IL-6) plays a central role in CRS pathophysiology [3] and has been used as an inflammatory biomarker. Tocilizumab, an IL-6 receptor blocker, is the first-line treatment for grade ≥2 CRS [4]. However, its administration leads to paradoxical increases in circulating IL-6 levels due to receptor saturation, rendering post-treatment values unreliable for clinical interpretation [5,6]. This creates an unmet need for additional dynamic and treatment-independent biomarkers of systemic inflammation.

Serum amyloid A (SAA) is a small, highly inducible acute-phase apolipoprotein produced mainly by hepatocytes in response to proinflammatory cytokines, including IL-1β, IL-6, and TNF-α [7]. Its serum concentration can increase by over 1000-fold within 24 h of acute inflammatory stimulation and decline rapidly upon resolution [8]. Beyond its role as a biomarker, SAA actively participates in immune regulation: it binds bacterial components such as LPS, promotes phagocytosis, and engages innate immune receptors—including toll-like receptors (TLR) 2, TLR4, and Formyl Peptide Receptor 2 (FPR2)—to amplify inflammatory responses [9]. In early-onset infections, SAA has shown high sensitivity—up to 92%—and faster response kinetics compared to C-reactive protein (CRP) [10]. Moreover, SAA has demonstrated superior diagnostic performance compared to CRP in various systemic inflammatory diseases in mammals’ models, highlighting its cross-species reliability as a sensitive inflammatory marker [11].

Notably, hepatic-derived SAA predominantly associates with high-density lipoproteins (HDL) in circulation, contributing to systemic lipid transport and inflammatory modulation. In contrast, SAA synthesized by activated macrophages and inflamed peripheral tissues tends to remain unbound and locally active, exerting direct effects on immune cell signaling within the inflammatory microenvironment [9].

Moreover, SAA can also be a compound of the neoplastic inflammatory burden. In patients with solid tumors, including uterine serous papillary carcinoma and breast cancer, elevated serum levels of SAA have been associated with disease progression and tumor stage, suggesting its potential as a tumor-associated inflammatory biomarker [12,13].

Similarly, in veterinary oncology, SAA has been proposed as a prognostic and monitoring tool for lymphoproliferative malignancies. In cats with gastrointestinal or multicentric lymphoma, SAA levels were significantly elevated at diagnosis and responded dynamically to treatment, with higher values correlating with disease severity and outcome [14,15].

However, the behavior and diagnostic potential of SAA in the setting of CAR-T-related inflammation, particularly CRS, remains underexplored.

In this study, we investigated the kinetics of SAA in adult patients receiving CAR-T cell therapy for LBCL and explored its association with early inflammatory toxicity. In addition, the role of SAA in the lymphoma-associated inflammatory status before CAR-T was also analyzed.

## 2. Materials and Methods

This retrospective observational study included all consecutive adult patients (age > 18 years) with LBCL who received CAR-T cell therapy at our institution between May 2023 and January 2025. To obtain a homogeneous population, we excluded patients diagnosed with B-cell acute lymphoblastic leukemia (*n* = 7), mantle cell lymphoma (*n* = 9), and follicular lymphoma (*n* = 7).

The data cut-off was set at January 2025 to ensure full documentation of early toxicities (up to day +30) and assessment of disease status by PET-CT within three months (M3) for all enrolled patients.

Peripheral blood (PB) samples were collected at predefined timepoints: before lymphodepletion, on the day of CAR-T cell infusion (day 0), and every other day through day +11. At each timepoint, complete blood counts and a panel of acute-phase proteins (APPs) were evaluated. These included CRP, Interleukin-6 (IL-6), procalcitonin (PCT), ferritin, fibrinogen (Fbg), soluble suppression of tumorigenicity 2 (sST2), and soluble urokinase plasminogen activator receptor (suPAR). SAA was measured at the same timepoints.

The modified endothelial activation and stress index (m-EASIX) was calculated prior to lymphodepletion [16]. Disease status was assessed by PET-CT within one month prior to CAR-T infusion and at one (M1), three (M3), and six (M6) months post-infusion, or in the event of suspected clinical progression.

CRS and immune effector cell-associated neurotoxicity syndrome (ICANS) were graded according to ASTCT consensus criteria [4]. Patients were stratified by maximal CRS grade during follow-up. ROC analyses paired SAA levels at predefined timepoints with same-day CRS grades.

Continuous variables were expressed as medians with interquartile ranges (IQRs), and categorical variables as absolute numbers and percentages. Group comparisons were performed using the Mann–Whitney U test or Kruskal–Wallis test for continuous variables, and Fisher’s exact test or Chi-squared test for categorical variables. Correlations were analyzed using Spearman’s rank correlation coefficient. Associations between variables and selected outcomes were evaluated using backward stepwise logistic regression. Statistical analyses were conducted using NCSS 2020 software.

This study was conducted in accordance with the principles of the Declaration of Helsinki and approved by the local ethics committee (ID 4879, Prot. 0020777/22, Amendment 09/2024). Written informed consent was obtained from all participants for the anonymous use of their data.

## 3. Results

This pilot analysis included 43 patients who all received axicabtagene ciloleucel. Patient characteristics are summarized in Table 1.

The median baseline SAA level was 13.6 mg/L (range, 3.5–692 mg/L), exceeding the upper limit of normal (ULN) of 6.4 mg/L in 33 (77%) cases. Baseline SAA values seemed slightly higher in males (median 40.45 vs. 11.55 mg/L) and in patients aged more than 60 (median 19.6 vs. 9.6 mg/L), despite no statistical significance being observed, *p* = 0.085 and *p* = 0.161, respectively).

**Figure 1:**
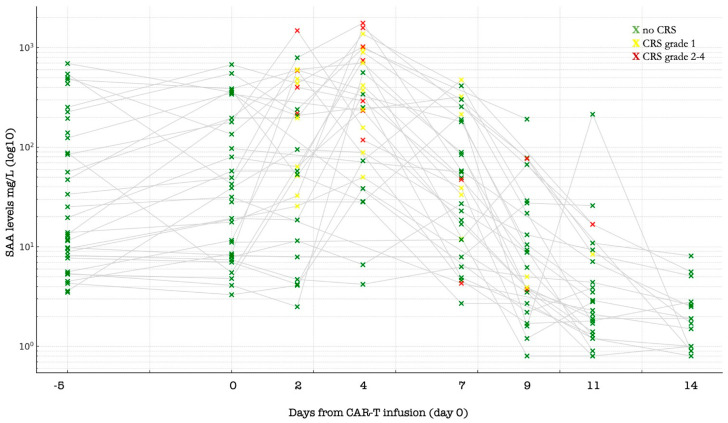
Higher SAA levels in patients with active CRS compared to patients not experiencing active CRS at specific timepoints after CAR-T infusion.

Starting from the initiation of lymphodepletion, SAA levels exhibited a wave-like kinetic profile. Levels increased after CAR-T infusion, peaking at day +4 (median 270 mg/L, range 4.2–1760), then declined progressively from day +7 (median 33.3 mg/dL), stabilizing below the ULN by day +11 (median 3.5 mg/dL) (Figure 1). Paired analysis revealed that SAA levels on day 0 (infusion day) were significantly higher than on day +11 (Wilcoxon signed-rank test, *p* = 0.006).

**Figure 2:**
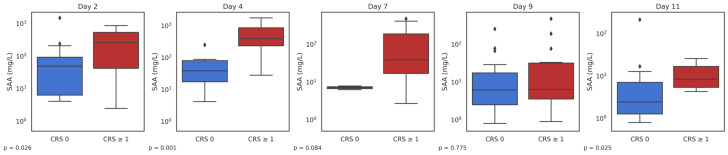
SAA measured at different timepoints was higher in patients experiencing concomitant CRS compared to patients with no active CRS at that timepoint.

Next, we examined the relationship between SAA and toxicities related to CAR-T cell therapy. Cytokine release syndrome was observed in 95% patients (41/43), overall, being of grade 1, 2, or 3, 4 in 39%, 54%, 5%, and 2%, respectively. Baseline SAA levels were higher in patients with any CRS compared with patients with no CRS at any timepoint, but the small size of the group that never experienced CRS makes a direct comparison unlikely. After this, we analyzed at day 2, 4, 7, 9, and 11 if SAA measured at that timepoint was different among patients actively experiencing CRS in that same day: in day 2, 4, 7, 9, and 11, CRS was ongoing in 56%, 77%, 91%, 39%, and 16% of patients, respectively, with levels of SAA significantly different at day 2 (49.2 vs. 260.0, *p* = 0.026), day 4 (374.0 vs. 55.8 mg/L, *p* = 0.001), and day 11 (8.4 vs. 2.4 mg/L, *p* = 0.025) (Figure 2).

Notably, the two patients who did not develop CRS had significantly lower baseline SAA levels on day 5 (median 4.05 mg/L, IQR 3.83–4.28) than the rest of the cohort (median 16.75 mg/L, IQR 8.73–128.0; Mann–Whitney U test, *p* = 0.039). However, their SAA levels on day 0 (median 23.75 mg/L, IQR 16.13––31.38) were not significantly different from those of patients who developed CRS (median 49.4 mg/L, IQR 10.3–157.0; *p* = 0.51). Peak SAA levels did not differ significantly between CRS subgroups when comparing patients with CRS grades 1–2 (*n* = 38; median 416.0 mg/L, IQR 118.0–1020.0) and patients with CRS grades 3–4 (*n* = 3; median 550.0 mg/L, IQR 543.0–743.0; Mann–Whitney U test, *p* = 0.30).

Restricting the analysis to post-infusion measurements (≥ day 0), SAA peaked around CRS onset (median peak–onset difference 0 days; range −9 to +7). There were no significant differences based on CRS grade (1–2 vs. 3–4; *p* = 0.88) or CRS duration (≤5 vs. >5 days; *p* = 0.53).

The median SAA values for CRS grades 0, 1, 2, and 3 were 9.2, 65.0, 202.5, and 47.3 mg/L, respectively. These values were calculated using the recorded value for each patient from days 0 to 11 after infusion.

ICANS was observed in 35% patients, being of grade 1, 2, and 3, 4 in 53%, 27%, 13% and 7%, respectively. No significant association was found between SAA levels and the occurrence of ICANS (*p* = 0.664, Mann–Whitney U test).

**Figure 3:**
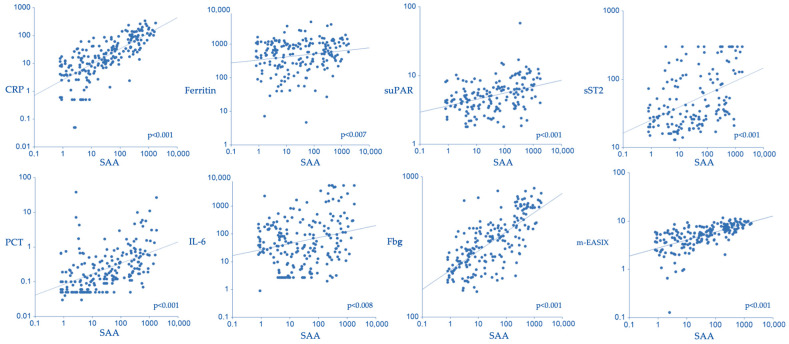
SAA correlates with acute-phase and endothelial activation markers. Legend: Correlation between serum amyloid A SAA (mg/L) and CRP C-reactive protein (mg/L), ferritin (ng/mL), suPAR soluble urokinase plasminogen activator receptor (ng/mL), sST2 soluble suppression of tumorigenicity 2 (ng/mL), PCT procalcitonin (ng/mL), IL-6 Interleukin-6 (ng/L), Fbg fibrinogen (ng/mL), and m-EASIX-modified endothelial activation and stress index (CRP [mg/L] × lactate dehydrogenase (LDH) [U/L]/platelets [×10^9^/L]).

We then evaluated whether SAA correlated with other APPs during early CAR-T therapy. Spearman’s correlation analysis revealed strong direct associations between SAA and CRP (*p* < 0.001), suPAR (*p* < 0.001), sST2 (*p* < 0.001), fibrinogen (*p* < 0.001), procalcitonin (*p* < 0.001), ferritin (*p* = 0.007), and IL-6 (*p* = 0.008) (Figure 3).

A significant positive correlation was also observed between baseline SAA levels and the modified endothelial activation and stress index (mEASIX) (Spearman *p* < 0.001) (Figure 3).

Finally, we tested if baseline SAA could reflect tumoral inflammatory burden. At baseline, SAA seemed lower in patients with lower disease bulk before CAR-T (responders to bridging therapy) compared to those with active disease after bridging, but this difference did not reach statistical significance (49.4 vs. 41.3 mg/L, *p* = 0.498; Mann–Whitney U test). Baseline SAA was also associated with lactate dehydrogenase (LDH) above 250 ULN (35% vs. 65%), and, notably, baseline SAA was significantly higher in patients with elevated LDH compared to those with normal LDH (135.0 vs. 26.6 mg/L, *p* = 0.018). A similar trend was observed for baseline CRP, with higher median levels in patients with active disease (8.5 vs. 6.6 mg/L).

We then assessed whether baseline SAA levels were associated with treatment efficacy. Patients achieving a complete or partial metabolic response (CR/PR) at 3-month PET had significantly lower baseline SAA levels compared to those with stable or progressive disease (SD/PD) (10.6 vs. 113.7.0 mg/L, *p* = 0.024), despite exhibiting similar kinetic trends over time.

To further evaluate the predictive value of baseline SAA, we conducted ROC curve analysis. Baseline SAA demonstrated moderate discriminative performance for predicting non-response at 3-month PET, with an AUC of 0.77. An optimal cut-off of 47.02 mg/L yielded a sensitivity of 71% and a specificity of 83% (Figures S1 and S2).

Figure 1 shows serum amyloid A (SAA) Levels (logarithmic Y axis) after CAR-T Infusion according to CRS experienced on various timepoints. Patients’ timepoints are shown with a green, yellow, or red cross if CRS grade 0, 1, or 2+ was experienced at that timepoint. The figure helps to show that final SAA levels are far lower for most patients at the end of treatment than before, and that peak SAA is found when higher CRS is ongoing.

## 4. Discussion

SAA is an acute-phase reactant that can increase 100- to 1000-fold above baseline levels [17]. In our study, we confirmed a strong association between SAA and other APPs during the initial phases of CAR-T therapy, following peak-and-down kinetics (Figure 3). Clinically, higher SAA levels were observed in patients who developed any-grade CRS. Although IL-6 is widely used in clinical settings, its levels are often affected by a rebound after treatment with tocilizumab, limiting their interpretability [18,19,20]. In this setting, SAA emerges as a sensitive and dynamic biomarker of systemic inflammation during CAR-T cell therapy, as it is not directly targeted by anti–IL-6 receptor treatments. The limited sample size and occurrence of high-grade CRS (around 10% of all CRS) impaired our ability to determine whether SAA could predict higher-grade CRS. However, further studies may clarify this aspect. We also found a significant correlation between SAA levels and the modified endothelial activation score (mEASIX), a surrogate marker for endothelial activation. Elevated mEASIX indicates endothelial dysfunction and has been associated with CAR-T-related toxicities and outcomes [1].

Post-treatment SAA levels were generally lower than pre-treatment values, suggesting an association between SAA and pre-existing neoplastic inflammation related to lymphoma. Furthermore, baseline SAA levels were significantly higher in patients with elevated LDH levels than in those with normal LDH levels, which supports the idea that SAA is linked to tumor burden or disease activity.

Based on the hypothesis that SAA is a marker of neoplastic inflammation, SAA levels were significantly higher in patients who failed to achieve a metabolic response at one and three months. These results suggest that SAA may serve as a surrogate marker of tumor burden and CAR-T cell therapy resistance, which is consistent with previous findings regarding solid tumors [10,11]. Similar associations have been described in veterinary oncology. For instance, cats with gastrointestinal or multicentric lymphoma had markedly elevated SAA concentrations at diagnosis, which decreased following treatment. Higher levels correlated with disease severity and clinical outcome [13,14]. While preliminary, these findings suggest a potential predictive role for SAA that warrants further validation in larger cohorts.

Collectively, the results suggest that SAA could be a useful biomarker indicating both inflammatory activity and early clinical outcomes in patients undergoing CAR-T cell therapy. Our study is limited by its single-center, retrospective-designed and small cohort size, which limits generalizability. Nonetheless, the consistent associations across toxicity and efficacy endpoints justify further prospective validation. If confirmed, SAA could be incorporated into pre-infusion risk models such as InflaMix [21] and early CRS-monitoring strategies, particularly in settings where IL-6 measurement is confounded or unavailable.

## 5. Conclusions

SAA levels demonstrated clear acute-phase-like kinetics following infusion of CAR-T, and strongly correlated with several acute-phase proteins and clinical development of CRS. Moreover, baseline SAA levels could mirror inflammatory neoplastic burden, and can be associated with treatment response. This is, to our knowledge, the first report evaluating serum amyloid A in the setting of CAR-T therapy for hematologic malignancies.

## Figures and Tables

**Table 1:** Baseline characteristics of the study population.

Characteristics	Population (*n* = 43)
Male	*n* = 28 (65%)
Female	*n* = 15 (35%)
Age, median (range)	59 (23–74)
LBCL subtype	DLBCL NOS *n* = 32 (74%)
	EBV + DLBCL *n* = 5 (12%)
	tFL *n* = 4 (9%)
	PMBCL *n* = 2 (5%)
Stage	Stage II *n* = 5 (12%)
	Stage III *n* = 3 (7%)
	Stage IV *n* = 35 (81%)
LDH > ULN (≈250 U/L)	*n* = 15 (35%)
IPI at diagnosis	IPI 1 *n* = 7 (16%)
	IPI 2 *n* = 12 (28%)
	IPI 3 *n* = 13 (30%)
	IPI 4 *n* = 7 (16%)
	IPI 5 *n* = 4 (9%)
Number of prior therapy lines, median (range)	2 (1–4)
Disease status before CAR-T	CR *n* = 6 (14%)
	PR *n* = 10 (23%)
	SD *n* = 11 (26%)
	PD *n* = 16 (37%)
CAR-T product	Axicabtagene ciloleucel *n* = 43 (100%)

Legend: LBCL, large B-cells lymphoma; DLBCL, NOS diffuse large B-cell lymphoma not otherwise specified; EBV+DLBCL, Epstein–Barr virus-positive diffuse large B-cell lymphoma; tFL, transformed follicular lymphoma; PMBCL, primary mediastinal large B-cell lymphoma; LDH, lactate dehydrogenase; IPI, international prognostic index; CR, complete response; PR, partial response; SD, stable disease; PD progressive disease.

## Data Availability

Data available upon request addressed to the corresponding author.

## Supplementary Materials

The following supporting information can be downloaded at: https://www.mdpi.com/article/10.3390/cancers17193184/s1, Figure S1: Baseline Serum Amyloid A (SAA) is higher in patients who achieve response to CAR-T at the evaluation at 3 months after CAR-T infusion (M3)_ T-test and ROC analysis. Figure S2: ROC ANALYSIS comparing acute phase-protein- namely ferritin, interleukin-6 (IL-6), serum amyloid A (SAA), and C-reactive protein (CRP)- and their ability to identify active CRS (grade 0 vs. any grade). CRP and SAA are the best predictors with AUC of 79% and 75%, respectively.
