# Supplementary material for: Tracking Inflammation in CAR-T Therapy: The Emerging Role of Serum Amyloid A (SAA)
Source: Cancers (Basel). 2025 Sep 30;17(19):3184. doi: 10.3390/cancers17193184 (PMC12523542; doi:10.3390/cancers17193184)
Supplement: Supplementary file 1 [file cancers-17-03184-s001.zip › cancers-3787902-supplementary.pdf]

cancers

Supplementary Materials: Tracking Inflammation in CAR-T Therapy: The Emerging Role of Serum Amyloid A (SAA)

Ilaria Pansini , Eugenio Galli, Alessandro Corrente, Marcello Viscovo, Silvia Baroni, Nicola Piccirillo, Patrizia Chiusolo, Federica Sorà and Simona Sica

Figure S1. Baseline Serum Amyloid A (SAA) is higher in patients who achieve response to CAR-T at the evaluation at 3 months after CAR-T infusion (M3)_ T-test and ROC analysis.

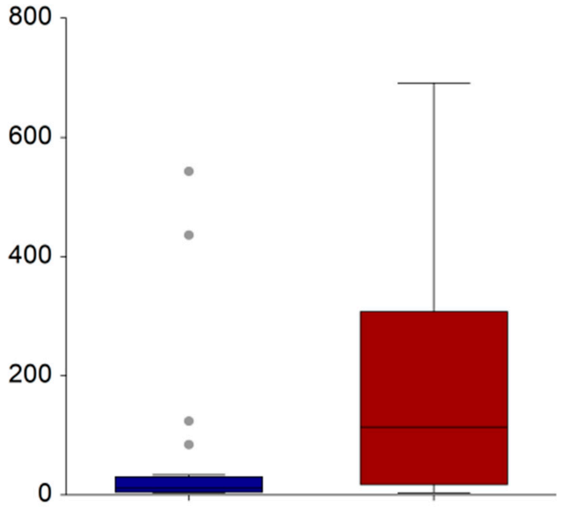

X AXIS: responders (blue) vs non-responders

Y AXIS: baseline SAA levels (mg/L)

				Z-Value	Upper	
		**Standard**	**to Test**	**1-Sided**	**95% Confidence Limits**	
**Criterion**	**AUC**	**Error**	**AUC > 0,5**	**P-Value**	**Lower**	**Upper**
SAA	0,7708	0,0878	3,084	0,0010	0,5358	0,8950

Best cut-off value: 47.02 mg/L

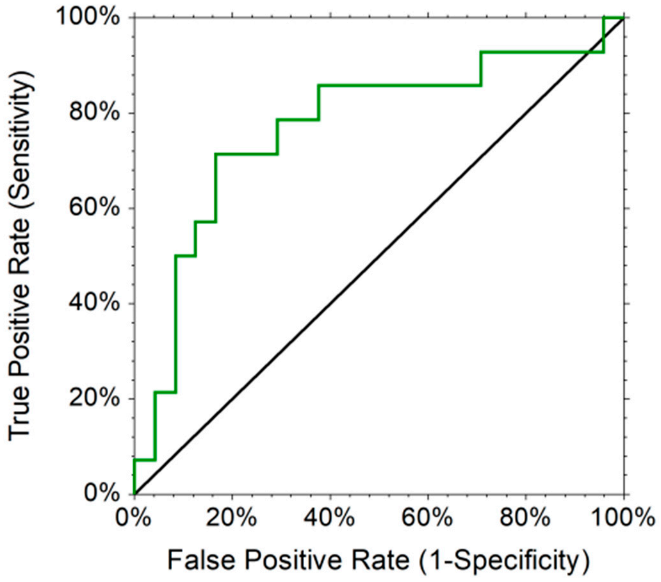

Figure S2. ROC ANALYSIS comparing acute phase-protein- namely ferritin, interleukin-6 (IL-6), serum amyloid A (SAA), and C-reactive protein (CRP)- and their ability to identify active CRS (grade 0 vs any grade). CRP and SAA are the best predictors with AUC of 79% and 75%, respectively.

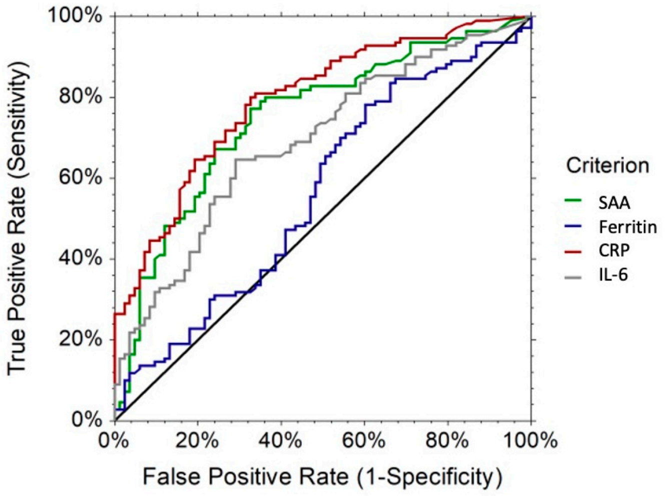

Criterion	AUC	Standard Error	Z-Value to Test AUC > 0,5	Upper 1-Sided P-Value	95% Confidence Limits Lower	Upper
SAA	0,7504	0,0358	7,004	0,0000	0,6715	0,8125
Ferritin	0,5645	0,0424	1,521	0,0641	0,4757	0,6418
CRP	0,7939	0,0319	9,207	0,0000	0,7224	0,8486
IL-6	0,6914	0,0380	5,043	0,0000	0,6095	0,7587
